# The Impact of Landfill Operations on Borehole Water Quality

**DOI:** 10.1155/2024/8899070

**Published:** 2024-11-04

**Authors:** Lyndon N. A. Sackey, Rita Kwablah, Lawrencia S. Y. Agyemang

**Affiliations:** Department of Environmental Science, Kwame Nkrumah University of Science and Technology, Kumasi, Ghana

**Keywords:** bacteria, groundwater, heavy metals, landfill site, water quality

## Abstract

Water is crucial for life, and although groundwater is considered safe, it can degrade due to inadequate source protection and inefficient resource management. The aim of the study was to assess the impact of landfill operations on water quality from selected landfill sites. Spectrometry analytical techniques were used to assess the physicochemical parameters of the samples. The study found that while Salmonella was absent in most samples, *E. coli* and total coliforms were present, making the water unsafe for domestic use. Although turbidity, pH, temperature and other parameters were within acceptable GSA/WHO levels, total coliform and *E. coli* raise concerns about faecal contamination and potential health risks. Cadmium was absent in all samples, but chromium, lead, arsenic and mercury were detected. These heavy metals could pose health hazards to consumers. The samples showed no risk of noncarcinogenic and carcinogenic risk to consumers, but then there is a potential health risk to consumers over time due to bioaccumulation. Regular monitoring and periodic assessments are recommended to ensure water safety. Proper waste management practices in landfill areas can minimize potential impacts on water quality, highlighting the need for continuous monitoring and assessment. The municipal authorities can inform residents of the situation and put in policies to protect human health. The results could help residents know the level of contamination of water from their boreholes and the necessary precautions to take to reduce their health impact. A treatment system can be developed in detail to their peculiar need.

## 1. Introduction

All living organisms require a lot of water; about two-thirds of the human body is water. Musie and Gonfa [1] reported that 97% of the earth's water is found in the oceans and 3% of the earth's water is fresh. Groundwater and surface water reservoirs are significant freshwater resources [2]. Currently, two billion people are without access to clean drinking water in the world [3]. Drinking unsafe, polluted water has several negative effects that are yet to be fully comprehended [4]. Exposure to these pollutants through contaminated borehole water can result in various adverse health effects, including cancer [5], renal damage, hypertension, and neurological disorders [6]. Due to the periodic water shortage that has been affecting most areas of cities, groundwater is increasingly being used as a source of drinking water for residents of both rural and urban populations [7]. According to Reno [8], waste is a material that affects the world and is mostly produced by human activities such as manufacturing and processing of consumption commodities. One of the main issues affecting the environment and public health in large cities in developing nations is the management of municipal solid waste (MSW) [9].

Solid waste management is a significant issue in many countries, especially in emerging ones with rapid population growth, often disposed of in nonengineered or engineered landfills [10, 11]. This is a primary challenge faced by several developing nations, including Ghana, due to its inability to manage municipal solid garbage in an environmentally friendly manner. Because of the potential harm of landfill leachate pollution to human health, its awareness has intensified [9]. According to Gonzalez-Valencia, Magana-Rodriguez, Cristóbal, and Thalasso [12], landfills are the least expensive, easiest and most cost-effective techniques for managing solid waste. Landfills pose environmental hazards due to improper handling, leachate and gas composition. Waste disposal exposes it to erosion, groundwater infiltration and water percolation [10, 11]. Because of its many benefits, including its low technological constraints and economic efficiency, emerging nations in particular employ landfills as their principal option for getting rid of harmless solid waste [12].

Groundwater pollution is a global concern due to human intervention, inappropriate agricultural drainage, untreated industrial effluents and leachate from landfill sites [13]. Waste management in Ghana is challenging due to industrialization, urbanization and anthropogenic activities. Population growth and inadequate water supply increase demand for groundwater in Kpone and Nsumia. Leachate leakage to groundwater is possible due to nonengineered landfill sites. Assessing water quality in boreholes near landfills is crucial.

Groundwater faces significant challenges due to degradation caused by human intervention and inappropriate agricultural drainage [13]. Anthropogenic sources such as untreated industrial effluents, clumsy disposal of household waste and runoff from agriculture are the prime contributors to surface and underground water pollution and water quality deterioration [14, 15]. Leachate from landfill sites can result in groundwater pollution which is an overwhelming problem of concern around the world. Waste management is a challenge in most developing countries, and Ghana is no exception. Industrialization, urbanization and other anthropogenic activities contribute to rising waste generation. A high population growth rate is at the bedrock of these factors mentioned. This also has increased demand for water supply, which is not being met by the state. Due to inadequate state water supply, groundwater is used extensively in Kpone and Nsumia through wells and boreholes. Leachate leakage to groundwater is possible because most landfill sites in Ghana are not engineered. However, to understand the level of contamination of leachate to groundwater, there is a need to assess the boreholes situated close to landfills. Hence, there is a need to determine the water quality of boreholes situated close to these landfill sites. The results obtained from this research will be relevant in the future management of engineered landfills and also for stakeholders interested in water and sanitation issues. The findings might contribute to and serve as a foundation for future research on the usage and treatment of groundwater or borehole water around a landfilled site in the context of how to manage the site to prevent leachate leaching into the environment.

## 2. Materials and Methods

### 2.1. Study Site

**Figure 1:**
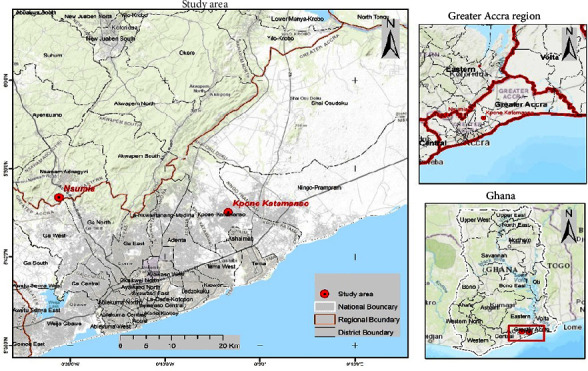
Map of study areas indicated with red circle in the map.

The study area was Kpone and Nsumia located in the Greater Accra. Kpone is a town located in the Greater Accra Region of Ghana. It is situated along the eastern coast of the country, which falls within the Kpone Katamanso Municipal District (Figure 1).

Kpone is primarily an industrial and residential area, with various industrial establishments and residential communities. It is known for hosting several industries, including manufacturing, construction, and oil and gas-related activities. The area has seen significant development and growth in recent years, attracting local and international businesses. Kpone landfill site is located about 2 km off Kpone Police Barrier on the main Accra-Aflao Road and has good all-weather accessibility. The region around Kpone is characterized by a tropical climate, with high temperatures and distinct wet and dry seasons.

Nsumia is a rural area in Ghana. Nsumia is situated northwest of Medie, and south of Nsawam, surrounded by several other towns and villages, including Amasaman, Pokuase and Oduman. The area is characterized by a mix of urban and rural settings, with ongoing development and infrastructure improvements.

### 2.2. Sample Collection

Ten samples were randomly collected from boreholes in the selected locations around the study area Kpone and Nsumia, for the analysis. The distances of the boreholes from the landfill sites were also considered during the sample collection. The samples were collected from the boreholes using cleaned receptacles. They were kept in a presterilized 1.5 L of polyethylene bottles and were transported to the laboratory in an ice chest at a temperature of 4°C.

Table 1 provides information on the various sampling points of the study sites.

### 2.3. Analytical Procedure

The parameters such as temperature, pH, salinity, total dissolved solids and electrical conductivity (EC), dissolved oxygen (DO), total suspended solids (TSS), chloride, total hardness, total coliforms, *E. coli* and Salmonella, phosphate, sulphate, iron, nitrate–nitrogen, nitrogen–ammonia and turbidity were analysed using various analytical methods.

The colour, total hardness, calcium and magnesium were determined using the standard method. All the analyses were done in triplicate.

### 2.4. Determination of pH, Conductivity, Turbidity, TDS and Temperature and DO

The pH, conductivity, turbidity and temperature metre were measured using the Palintest Micro 800 Multi. The WTW Oxi 3205 oximeter was used to measure the DO in the samples.

### 2.5. Determination of TSS

A 100-mL portion of a thoroughly mixed sample was passed through a standard glass-fibre filter that had been previously weighed. The solid residue captured by the filter was subsequently subjected to drying in an oven within the temperature range of 103°C–105°C for a duration of 1 h. It was cooled in a desiccator and then weighed. The additional weight acquired by the filter corresponds to the measurement of TSS.

#### 2.5.1. Calculation

The TSS was calculated using the formula as shown in the following equation:

(1) $$\begin{array}{c} \frac{\text{mg total suspended solids}}{L}=\frac{\left.\left(\left(A-B\right)\text{mg}\times 1000\right)\right)}{\left(\text{sample volume},\text{mL}\right)}, \end{array}$$

where *A* = weight of filter + dried residue (mg) and *B* = weight of filter (mg).

### 2.6. Determination of Chloride

Chloride presence was ascertained using the argentometric method [16]. A known sample volume (50 mL) of wastewater had 1.0 mL of K_2_CrO_4_ added to it. This solution was titrated with a standard AgNO_3_ titrant until it reached a pinkish-yellow endpoint. The same procedure was carried out with an equal volume of distilled water to establish the blank. The concentration of chloride was then determined using the following equation:

(2) $$\begin{array}{c} \text{mg}\frac{{\text{Cl}}^{-}}{L}=\left(\frac{\left(A-B\right)\times N\times 35450}{\text{sample mL}}\right), \end{array}$$

where *A* = mL titration for sample, *B* = mL titration for blank, and *N = *normality of AgNO_3_ (0.0141M).

### 2.7. Pollution Indices

#### 2.7.1. Contamination Factor (CF)

The CF and pollution load index (PLI) were calculated for each borehole to evaluate the degree of heavy metal contamination. The CF was determined using the following equation:

(3) $$\begin{array}{c} \text{CFi}=\frac{\text{Ci}}{\text{Bi}}, \end{array}$$

where Ci and Bi are the measured concentration and the background value of metal *i*, respectively. A CF below 1 indicates low contamination.

#### 2.7.2. PLI

The PLI was computed using the following equation:

(4) $$\begin{array}{c} \text{PLI}=\left(\text{CF}1\times \text{CF}2\times \text{CF}3\times \ldots \times \text{CF}n\right)\frac{1}{n}, \end{array}$$

where *n* is the number of heavy metals involved.

#### 2.7.3. Health Risk Assessment

The potential health risks of heavy metal contamination were evaluated by calculating the target hazard quotient (THQ), Hazard Index (HI) and cancer risk (CR) for each sample. The THQ was computed using equation (5).

### 2.8. Chronic Daily Intake (CDI)

(5) $$\begin{array}{c} \text{THQ}=\frac{\text{CDI}}{\text{RfD}}, \end{array}$$

where CDI represents the estimated intake of heavy metals per kilogram of body weight, and RfD stands for the oral reference dose of the heavy metals (expressed in mg/kg/day) through the oral exposure route. The respective RfD values for Cd, Cr, Hg, Pb and As are 0.0005, 0.0003, 0.0001, 0.0003, 0.0004, 0.0085 and 0.0003 mg/kg/day [17].

CDI was determined using the following equation:

(6) $$\begin{array}{c} \text{CDI}=\frac{C\times \text{IRi}\times \text{EFi}\times \text{EDi}}{\text{BWi}\times \text{AT}}, \end{array}$$

where *C* is the concentration (mg/L) of heavy metal in samples, IRi is the ingestion rate for adults and children consumers (adult and children 12 and 11 mL/kg-day, respectively), EFi is the exposure frequency (350 days/year for both age groups), EDi is the exposure duration (for adults and children 70 and 6 years, respectively), BWi is the body weight (for children and adults 20 and 70 years, respectively), and AT is the average time lifespan (EF × ED) (adults and children 25,550 and 2190 days, respectively) [18]. A THQ exceeding 1 indicates potential health concerns.

To determine the overall total target hazard quotient (TTHQ) for all the heavy metals, the summation of individual THQi values for each heavy metal was computed using the following equation:

(7) $$\begin{array}{c} \text{TTHQ}=\overset{n}{\underset{i=1}{\sum }}\text{THQi}. \end{array}$$

The CR was derived using the following equation:

(8) $$\begin{array}{c} \text{CR}=\text{CDI}\times \text{CSF}\times \text{ADAF}, \end{array}$$

[19] where CSF represents the cancer slope factor (expressed in kg/day/mg) and ADAF is the age-dependent adjustment factor (for adults and children 1 and 3 values) [18]. The calculated CR value was subsequently compared to the maximum acceptable risk threshold [17, 20]. CSF for As is 1.5 kg.day/mg, Pb is 8.5 × 10^−3^ kg.day/mg, Cr is 0.5 kg.day/mg, Cd is 15 kg.day/mg [21], and Hg was not available. In this study, the estimated CR value was compared with the maximum acceptable risk suggested by the USEPA, which is ≤ 1E − 6 [17].

### 2.9. Determination of Total Coliforms, *E. coli* and Salmonella

The membrane filter technique using Chromocult coliform agar was used to determine the presence of bacteria [22, 23].

### 2.10. Spectrophotometric Analysis

A DR/3900 Spectrophotometer was used in determining the concentration of iron, phosphates, sulphates, nitrate–nitrogen–nitrogen and nitrogen–ammonia. The PhosVer 2 (ascorbic acid) method was used to determine phosphate. Sulphate was determined using the SulfaVer 4 method. For iron, the FerroVer method was used in the analytical analysis. Nitrate–nitrogen was analytically determined using the cadmium reduction method. The salicylate method was used to analytically determine the nitrogen–ammonia in the sample. Some selected heavy metals (Cd, Cr, Pb, As and Hg) were quantified using microwave plasma atomic emission spectroscopy (MP-AES).

### 2.11. Statistical Analysis

Data were organized using Microsoft Excel 2016 and statistically analysed using analysis of variance (ANOVA) and Pearson correlation to establish relations between the variables.

## 3. Results and Discussion

### 3.1. Physicochemical Quality Characteristics

Turbidity values ranged from 0.22 to 2.08 NTU, and colour values ranged from 0.00 to 22.00 Pt/Co. Both turbidity and colour values were within the acceptable limits defined by the World Health Organization (WHO) and the Ghana Standard Authority (GSA). This indicated the water was relatively clear and free from visible particulates and discoloration. The results of the chemical analysis of the water samples of the study area are presented in Table 2. It also indicates that less silt, clay and organic matter percolated from the landfill sites into the boreholes. However, continuous consumption can cause health impacts. This could be due to the filtration of organic matter and other contaminants such as heavy metals and ammonia nitrogen compounds, as well as emerging pollutants such as pharmaceuticals, plasticizers and per- and polyfluoroalkyl substances (PFAS) as the leachates seep through the sand and rocks [24]. Amoako, Karikari and Ansa-Asare [25] reported similar results: Groundwater quality near a dump site at Kwadaso Sub-Metro in the Ashanti Region, Ghana, had low turbidity and pH within the WHO guideline limits. The pH was within the permissible limit, and this indicates that there might not be contamination of dissolved organic matter, inorganic macrocomponents, heavy metals and xenobiotic organic compounds of the leachate leaching into the boreholes or that the levels of leachate percolation are not yet at levels that could cause harm to human health [26]. The similarities in the results can be attributed to a combination of geological and environmental factors. This could affect the water quality and hence might cause health problems for the consumers such as abdominal pain, diarrhoea, vomiting, confusion, drowsiness, seizures, bloody diarrhoea, dehydration and renal failure. The high presence of NO_3_^−^ in some sampling sites could be due to nitrogen-based fertilizer and their distance from the site. The potential health effects of high nitrate levels in drinking water include reproductive problems, methemoglobinemia, colorectal cancer, thyroid disease and neural tube defects [27]. Infants are especially at risk for methemoglobinemia (‘blue baby' syndrome).

While some sampling locations have higher values than the GSA limit of 1000 µS/cm, assessing the specific nature of these dissolved substances is essential. The conductivity levels at ABH 3, EH 1 and DH 2 were higher than the other sampling points. The elevated conductivity could indicate the presence of dissolved ions and contaminants such as household products and road runoff, and the presence of these pollutants might affect domestic use [6]. Several sampling points were within the GSA limit, suggesting suitable groundwater quality for domestic use and household activities. Usually, high levels of conductivity affect the taste of water [28]. However, the proximity to the landfill raises concerns about the potential contamination and the influence of landfill-related contaminants on water quality. The sampling locations with higher conductivity levels require careful monitoring and investigation.

The total dissolved solid (TDS) concentrations varied from 241.50 to 1987 mg/L with some of the samples exceeding the GSA standard of 1500 mg/L. TDS is a comprehensive measure of water's dissolved substances, encompassing various ions, minerals and trace elements. Elevated TDS can be caused by natural sources like geology or human activities like agricultural runoff and industrial discharges [29]. High TDS may affect the taste of the water and harm aquatic life [30].

The DO value ranged from 5.50 to 7.50 mg/L. The desired requirement of DO concentrations in water is 6 mg/L for drinking water, 4–5 mg/L [31]. Increased levels of DO can enhance the taste of water and might be the favoured by consumers. The insufficient penetration of light into the water could be a leading cause for these lower oxygen concentrations. Additionally, the impact of water movement, which could be influenced by lifting or other factors, may not significantly contribute to the observed variations.

The salinity values ranged from 15.45 to 202.44 mg/L, within the acceptable GSA limit of 250 mg/L. The samples from EH 1, ABH 3, BSC Gate 5 and AJ 3 exhibit relatively higher salinity levels compared to the others; this could be possible due to the presence of higher concentrations of dissolved salts as a result of percolation of leachate into the boreholes. The samples with the elevated salinity have higher TDS and conductivity levels. It was reported that drinking water salinity is associated with hypertension and hyperdilute urine among Daasanach pastoralists in Northern Kenya, according to Rosinger et al. [32]. Hence, water consumption from the boreholes with high salinity can cause health problems.

The total hardness of the water from the samples ranged from 160 to 822 mg/L. The total hardness for the water samples from ABH3 and EH 1 recorded the highest value by exceeding the acceptable limit of GSA for the total hardness in water. Total hardness primarily results from calcium and magnesium ions in water. The samples with the elevated hardness level have higher levels of the dissolved ion concentrations. High total water hardness influences mortality, in particular, cardiovascular mortality. In addition, several epidemiological investigations have demonstrated the relation among risk for cardiovascular disease, growth retardation, reproductive failure and other health problems [33]. Also, hard water can interfere with the action of soaps and detergents and can result in deposits of calcium carbonate, calcium sulphate and magnesium hydroxide (Mg(OH)_2_) and forms a substance called ‘soap scum' which is a sticky residue that can be difficult to remove from surfaces. Hence, this will affect the use of the boreholes for domestic activities very difficult and expensive.

Dissolved ion concentrations were analysed, and the results are presented in Table 3.

Calcium and magnesium ions contribute to water hardness and play essential roles in various biological and chemical processes. The Ca^2+^ and Mg^2+^ levels in the samples were all within the GSA permissible limits, even though some samples had higher concentration levels. Water hardness influenced by Ca^2+^ and Mg^2+^ concentrations can impact the effectiveness of soap and detergents, and excessive levels can lead to scaling in pipes and appliances. These ions also influence water's buffering capacity, helping stabilize pH levels.

The sulphate levels were less than half of the acceptable GSA limits of 250 mg/L at all sampling locations, with DH 2 and EH 1 having a sulphate level of 103 mg/L and 110 mg/L, respectively (Table 3). Sulphate ions are commonly found in natural waters and can originate from the weathering of rocks and minerals and human activities. Sulphate salts can influence the taste and odour of the water and can react with other substances in the water to form insoluble precipitates. Monitoring sulphate concentrations is important for maintaining water quality and preventing taste and odour issues. Apau, Agbovi and Wemegah [34] reported that the boreholes in the Aburi Municipality of the Eastern Region of Ghana contain low sulphate levels, correlating with the current results. The sulphate concentration in both the borehole and spring water sources supplied to Robe Town, Oromia region, Ethiopia, was found to be within the permissible limit [35].

Nitrate–nitrogen ions are essential nutrients for plant growth, but excessive levels can lead to water pollution and health risks. NO_3_^−^ pollution often stems from agricultural runoff, sewage discharge and the use of nitrogen-based fertilizers. Groundwater can also be contaminated by sewage and other wastes rich in nitrates [36]. Most of the samples have higher values of nitrate ions exceeding the GSA recommended levels. This indicates that there is a possibility of leaching of leachate from the landfill sites into boreholes, and this could cause to babies and affect how blood carries oxygen and can cause methemoglobinemia.

Ammonia nitrogen ions are product of organic matter decomposition and are also released from industrial processes. The ammonia levels in water can be influenced by temperature, pH and the presence of nitrifying bacteria that convert ammonia into less toxic compounds. From Table 3, there were no traces of ammonia ion concentration in most of the samples except in EH 1, DH 2 and AJ 3. The samples with some ammonia concentration traces were still within acceptable limits. This indicates the leachate that possibly leached into the boreholes has less or no ammonia nitrogen ions because the materials deposited at the landfill sites contain no such chemicals.

### 3.2. Bacteriological Analysis of the Water Samples

The analysis of bacteriological water quality involves examining water samples to determine the bacterial population count and when necessary to identify the bacterial composition.  Table 4 presents the results for the water samples on biochemical oxygen demand, total coliform, Salmonella and *E. coli.*

The analysis results for total coliform bacteria ranged from 5 cfu/mL to 279 cfu/mL, indicating contamination in the water samples. The water sample from Greenwich International School (GREEN 4) recorded the highest contamination of total coliform, and EH 1 recorded the lowest. Most of the samples from Nsumia had a total coliform presence in the water, which can be attributed to the leachate from the landfill, which percolates into the groundwater. Some bacteria are released into the environment through human and animal waste disposal in open dumpsites. There were no Salmonella traces in any of the samples. Water contaminated with Salmonella can lead to gastrointestinal illnesses, including diarrhoea and fever, which can be severe in vulnerable populations such as children, the elderly and individuals with weakened immune systems. *E. coli* is a type of bacteria that can serve both as an indicator of water quality and as a pathogen with potential health risks when present in water sources. *E. coli* in some of the samples is of significant concern; its presence in the water is a strong indicator of faecal contamination, which may include harmful pathogens from human or animal waste. This indicates a high risk of contracting a water-borne illness when consumed. The presence of *E. coli* and coliforms indicates that the water is polluted, and so for it to be safe for drinking, filtration must be used to remove the bacteria from the water before use. Elevated levels of total coliforms in water samples can serve as an early warning sign of deteriorating water [37]. *E. coli* and coliform bacteria are commonly found in the intestines of warm-blooded animals, including humans. Contaminated water sources can result from improper disposal of human and animal waste, leading to the introduction of these bacteria into water bodies.

### 3.3. Heavy Metal Analysis of Water Samples

Cadmium (Cd) was not detected in all the tested samples. Chromium (Cr) had a mean value of 0.00092 mg/L, less than the WHO [38] standard value of 0.05. Lead (Pb) tested positive in all the sites (Table 5). An average value of 0.15 mg/L higher than the WHO [38] standard value of 0.01 was obtained. Arsenic (As) metals were recorded in all the samples tested. Compared to the WHO [38] value of 10 *μ*g/L, an average of 0.41 *μ*g/L was obtained. Mercury (Hg) had an average value of 0.02 *μ*g/L as compared to the WHO standard value of 0.001 *μ*g/L. Mercury was recorded at the following sample sites; ABH 3, DH2, ETH1, EH and GREEN 4 (Table 5). The existence of heavy metals in boreholes constitutes health challenges to humans and that might have significant poisonous effects and therefore should be treated before being used for drinking purposes.

A study by Nwoke and Edori [39] indicated a high concentration of heavy metals in borehole water from Ikono Urban, Ikono Local Government Area, Akwa Ibom State, Nigeria. Heavy metal contamination and risk assessment of borehole water within a landfill in the Nnewi Metropolis study suggest that the levels of heavy metals obtained were high, which was attributed to leachate percolation through the unlined refuse dump [40]. This is due to the infiltration of pollutants from the leachates because of its proximity to the dump site.

Generally, there is a strong correlation between the heavy metals detected in the various boreholes, indicating that an increase in any of the heavy metals will cause an increase in all the heavy metals detected except at KKC 6 and DH2 where the effect is contrary (-0.03) (Table 6) to the normal trend of relationship. This proves that there should be stringent monitoring of the boreholes to help control any possible increase of any of the heavy metals to protect human health.

### 3.4. Pollution Assessment

#### 3.4.1. CF

The CF and PLI were calculated to evaluate the degree of heavy metal pollution in the boreholes. As shown in Table 7, the CF values for all metals ranged from 0.00 to 3.5. Most boreholes were below the threshold of 1 denoting a low concentration of contaminants except ABH 3, DH2 and GREEN 2 which have CF of 3.5, 3.5 and 1.5 of mercury, respectively, denoting high mercury contamination within the boreholes [31]. This can affect the nervous system causing symptoms such as irritability, nervousness, changes in vision or hearing, and difficulties with memory [41]. The PLI, which combines the CF values, also suggests insignificant pollution, with the average across samples being 0.00024 (range 0.0001–0.0004) (Table 8). A similar study by Rahman et al. [31] on industrial effluent found Ni, Cd and Cr CFs indicating no contamination. The CF hierarchy showed Hg > As > Pb > Cd=Cr (Table 7), revealing Hg had the highest relative CF. However, the low mean CF and PLI values imply minimal pollution and contamination of the analysed borehole by the quantified heavy metals. The average PLI further showed this under the safe limit of 1 (Table 8). These results demonstrate that the examined boreholes have low metal pollution levels and are safe for consumers regarding contamination risks [31]. Even though the current assessment indicates safe borehole water samples, there is a need for continuous monitoring to prevent any possible surge that could affect human health. Furthermore, over time there could be possible health effects due to bioaccumulation.

### 3.5. PLI

#### 3.5.1. Health Risk Assessment

The potential health risks of heavy metals in the boreholes were evaluated by calculating the THQ, HI, and CR for each borehole location and age group (Tables 9, 10, 11, and 12). The THQ values for Cd, Cr, Pb, As and Hg remained within the safe limit of one in all samples for children, but for adults, it was Cd, Cr and Pb that remained in the safe limit [17]. However, THQs for As and Hg exceeded one in adults, indicating potential noncarcinogenic risks [42]. For instance, THQs up to 4.12 for As were detected in EH.

A similar study by Gwira et al. [41] also found high THQs, especially for AS and Hg in certain groundwater in Ghana. The THQ of the heavy metals was higher in adults than in children. The HI summed across metals surpassed one in adults and less than one in children for the eight borehole locations with KKC 6 having the highest HI (Tables 9 and 10). This indicates that adults have a high risk of being potentially affected when they ingest water from these boreholes, unlike children.

As per the guidelines set by the US Environmental Protection Agency [17], the acceptable limits for CR associated with carcinogenic metals such as arsenic (As), chromium (Cr), nickel (Ni) and lead (Pb) fall within the range of 1 × 10^−4^ to 1 × 10^−6^. All of the metals analysed were within acceptable limits in Tables 11 and 12 except Pb which was above concerning adults 1.31 × 10^6^–5.06 × 10^6^ (Table 11).

The CR values related to Pb for adults were a concern in all the boreholes examined. These values indicate a potential risk of cancer for consumers through prolonged exposure. A similar report by Gwira et al. [41] suggested a similar outcome where CR values for Pb were alarming in groundwater in Tarkwa a mining community in Ghana. Longer exposure duration could account for higher CR values in adults than in children [44]. Adults have more time to accumulate heavy metals in their bodies through various sources such as occupational exposure, environmental pollution and dietary intake [44]. Size and weight also play a role in these CR variations among adults and children.

This difference in body size can affect the dose of heavy metals received per unit of body weight. Children may receive a proportionally lower dose of heavy metals, reducing their CR compared to adults [45]. Implementing necessary control measures like Hazard Analysis and Critical Control Points (HACCP) is crucial to reduce the levels of these heavy metals in boreholes. Doing so could potentially decrease the CR from exposure to carcinogenic metals through the consumption of water from boreholes [18].

## 4. Conclusion

This study confirmed that the physicochemical properties of boreholes at different locations close to Kpone and Nsumia landfill sites in the Greater Accra Region of Ghana fell within the acceptable GSA and WHO limits. Turbidity values ranged from 0.22 to 2.08 NTU, and colour values were within the range of 0.00–22.00 Pt/Co. Both turbidity and colour values were within the acceptable limits defined by WHO and the GSA. This shows the water was relatively clear and free from visible particulates and discoloration. The pH was slightly acidic to neutral nature of the water and was below the GSA and WHO recommended. The comparative analysis of borehole quality near the Kpone and Nsumia landfill sites showed coliform bacteria contamination. Thus, making water from the sites unsatisfactory for drinking. Borehole samples collected closer to the landfills exhibit poorer physicochemical and microbiological water quality compared to those farther away. Also, the study confirmed the elevated levels of heavy metals such as mercury (Hg) and lead (Pb). The samples showed no risk of noncarcinogenic and carcinogenic risk to consumers, but then there is a potential health risk to consumers over time due to bioaccumulation. The Ghana Water Resources Commission, in collaboration with the Community Water and Sanitation Agency (CWSA) and the various district assemblies, should enforce regulations requiring monitoring and quality checks before boreholes and hand-dug wells are cited by private individuals. Regular monitoring and periodic assessments are recommended to ensure water safety. Operations and maintenance of the landfill must be done to reduce possible leaching of leachate to groundwater by ensuring continuous monitoring and assessment. The results could help residents know the level of contamination of water from their boreholes and the necessary precautions to take to reduce their health impact. A treatment system can be designed to meet their peculiar need. The government can connect the municipality to the main pipeline of the water treatment system to prevent any water-related disease.

## Figures and Tables

**Table 1:** Particulars of sample locations.

Location	Code	House number	Site	Treatment
Kpone	EH 1	Bro Eddie's House CJ250 China's Gh St.	In the house	No
Kpone	DH2	B/73 M3TSOKOPE	Outside	No
Kpone	ABH 3	AB House Adabranka	Outside	No
Nsumia	ETH 1	Mr. Ettey House	Inside the house	No
Nsumia	TGG 2	To God Be The Glory	In the house	No
Nsumia	AJ 3	Asante Junction	In the house	Yes
Nsumia	GREEN	Greenwich International School	In the house	Yes
Nsumia	BSC Gate 5	Blue Skies Composite Site Gate	In the house	Yes
Nsumia	KKC MAST 6	KK City Mast	In the house	Yes
Nsumia	KKC 7	KKC	Outside	No

*Note:* More samples were taken at Nsumia because Kpone is more urban and has water lines connected to the national system, unlike Nsumia, which is more rural without a water supply. Because of this, the distances of the boreholes at Kpone from the landfill sites are more distanced than that of Nsumia.

**Table 2:** Physicochemical parameters of water sample.

Samples	pH	Conductivity (*μ*S/cm)	DO (mg/L)	TDS (mg/L)	Total hardness (mg/L)	Salinity (mg/L)
EH 1	7.38	2670.00	6.00	1636	674	182.35
DH 2	7.38	2720.00	6.50	1669	382	159.17
ABH 3	7.80	3240.00	5.50	1987	822	202.44
ETH 1	6.49	590.00	7.10	362.00	290	30.92
TGG 2	6.90	410.00	7.35	251.20	166	18.54
AJ 3	6.40	781.00	6.90	478.60	290	63.36
GREEN 4	6.59	505.00	7.50	309.20	238	15.45
BSC GATE 5	6.57	538.00	7.70	330.00	180	55.63
KKC MAST 6	6.71	435.00	7.60	266.60	166	21.64
KKC 7	6.03	394.00	7.20	241.50	160	18.54

*Note:p* value < 0.05. There was a significant difference between the Kpone and the Nsumia landfill sites.

**Table 3:** Dissolved ion concentrations of the water samples.

Sample	Ca^2+^ (mg/L)	Mg^2+^ (mg/L)	SO_4_ (mg/L)	NO_3_-N (mg/L)	NH_3_
EH 1	128.26	70.01	110.00	28.80	0.10
DH 2	64.93	53.48	103.00	33.80	0.02
ABH 3	185.17	92.86	95.00	32.70	0.00
ETH 1	73.75	25.77	55.00	10.40	0.00
TGG 2	40.08	16.04	35.00	7.50	0.00
AJ 3	56.91	36.00	25.00	2.30	0.01
GREEN 4	78.56	10.21	38.00	13.80	0.00
BSC GATE 5	40.88	18.96	23.00	10.10	0.00
KKC MAST 6	50.50	9.72	24.00	17.90	0.00
KKC 7	35.27	17.50	26.00	26.00	0.00

*Note:p* value < 0.05. There was a significant difference between the Kpone and the Nsumia landfill sites.

**Table 4:** Bacteriological parameters of the water samples.

Site	BOD_5_ (mg/L)	Total coliform (cfu/mL)	Salmonella (cfu/mL)	*E. coli* (cfu/mL)
EH 1	3.0	5	0	0
DH 2	3.0	190	0	2
ABH 3	7.0	210	0	7
ETH 1	0.5	16	0	2
TGG 2	0.0	7	0	0
AJ 3	0.5	16	0	2
GREEN 4	0.0	279	0	0
BSC GATE 5	0.0	0	0	0
KKC MAST 6	0	0	0	0
KKC 7	0	106	0	0

**Table 5:** Heavy metal concentration in the water samples.

	Cadmium (Cd) (value ± SD)	Chromium (Cr) (value ± SD)	Lead (Pb) (value ± SD)	Arsenic (Ar) (value ± SD)	Mercury (Hg) (value ± SD)
ABH 3	0.01 ± 0.58	0.01 ± 0.06	0.27 ± 4.84	0.53 ± 0.71	0.07 ± 0.26
DH 2	0.01 ± 0.32	0.01 ± 0.03	0.24 ± 1.12	0.11 ± 12.33	0.07 ± 8.68
TGG 2	0.01 ± 1.37	0.01 ± 0.14	0.08 ± 6.24	0.44 ± 9.49	0.01 ± 5.79
ETH 1	0.01 ± 0.43	0.01 ± 0.26	0.09 ± 6.48	0.41 ± 7.67	0.01 ± 6.67
KKC 6	0.01 ± 0.3	0.30 ± 0.15	0.07 ± 9.57	0.48 ± 8.36	0.01 ± 11.99
KKC 7	0.01 ± 1.01	0.00 ± 0.13	0.08 ± 6.25	0.40 ± 15.35	0.01 ± 16.47
EH	0.01 ± 1.09	0.01 ± 0.05	0.23 ± 1.92	0.56 ± 4.88	0.02 ± 9.57
AJ 3	0.01 ± 0.45	0.01 ± 0.12	0.17 ± 4.2	0.43 ± 2.59	0.01 ± 7.03
BSC GATE 5	0.01 ± 0.54	0.01 ± 0.12	0.10 ± 5.76	0.44 ± 9.7	0.01 ± 7.93
GREEN 4	0.01 ± 0.64	0.01 ± 0.07	0.11 ± 6.47	0.36 ± 5.08	0.03 ± 7.33

*Note:p* value < 0.05. There was a significant difference between the Kpone and the Nsumia landfill sites.

**Table 6:** Correlation coefficient of the boreholes.

	*ABH 3*	*DH 2*	*TGG 2*	*ETH 1*	*KKC 6*	*KKC 7*	*EH*	*AJ 3*	*BSC GATE 5*	*GREEN GATE*
ABH 3	1									
DH 2	0.58	1								
TGG 2	0.94	0.2	1							
ETH 1	0.95	0.3	0.99	1						
KKC 6	0.67	−0.03	0.79	0.78	1					
KKC 7	0.94	0.29	0.99	0.99	0.7	1				
EH	0.99	0.49	0.97	0.97	0.71	0.9	1			
AJ 3	0.98	0.48	0.97	0.98	0.73	0.97	0.99	1		
BSC GATE 5	0.87	0.12	0.98	0.97	0.81	0.98	0.91	0.9	1	
GREEN GATE	0.87	0.12	0.98	0.97	0.80	0.97	0.91	0.91	0.99	1

**Table 7:** The contamination factor of the various boreholes.

Locations	Cadmium	Chromium	Lead	Arsenic	Mercury
ABH 3	0.01	0.01	0.01	0.04	3.50
DH 2	0.01	0.01	0.01	0.01	3.50
TGG 2	0.01	0.01	0.01	0.03	0.01
ETH 1	0.01	0.01	0.01	0.03	0.01
KKC 6	0.01	0.01	0.01	0.04	0.01
KKC 7	0.01	0.01	0.01	0.03	0.01
EH	0.01	0.01	0.01	0.04	1.00
AJ 3	0.01	0.01	0.01	0.03	0.01
BSC GATE 5	0.01	0.01	0.01	0.03	0.01
GREEN 4	0.01	0.01	0.01	0.03	1.50

**Table 8:** The pollution load index of the various boreholes.

Location	PLI
ABH 3	0.0004
DH2	0.0002
TGG2	0.0001
ETH 1	0.0001
KKC 6	0.0001
KKC7	0.0001
EH	0.0001
AJ3	0.0001
BSC GATE 5	0.0001
GREEN 4	0.0002

**Table 9:** THQ and HI of heavy metal contamination in samples (adult).

	Cd	Cr	Pb	As	Hg	HI
ABH 3	0.04	0.07	0.07	3.89	1.54	5.63
DH 2	0.04	0.07	0.06	0.81	1.54	2.53
TGG 2	0.04	0.07	0.02	3.24	0.22	3.60
ETH 1	0.04	0.07	0.02	3.02	0.22	3.38
KKC 6	0.04	2.20	0.01	3.53	0.22	6.02
KKC 7	0.04	0.07	0.02	2.94	0.22	3.30
EH	0.04	0.07	0.06	4.12	0.44	4.74
AJ 3	0.04	0.07	0.04	3.16	0.22	3.55
BSC GATE 5	0.04	0.07	0.03	3.24	0.22	3.60
GREEN 4	0.04	0.07	0.03	2.65	0.66	3.46
GTHQ	0.44	2.87	0.37	30.60	5.52	39.80

**Table 10:** THQ and HI of heavy metal contamination in samples (children).

Samples	Cd	Cr	Pb	As	Hg	HI
ABH 3	0.01	0.01	0.01	0.09	0.04	0.14
DH 2	0.01	0.01	0.01	0.02	0.04	0.06
TGG 2	0.01	0.01	0.01	0.08	0.01	0.08
ETH 1	0.01	0.01	0.01	0.07	0.01	0.08
KKC 6	0.01	0.05	0.01	0.08	0.01	0.14
KKC 7	0.01	0.01	0.01	0.07	0.01	0.08
EH	0.01	0.01	0.01	0.10	0.01	0.11
AJ 3	0.01	0.01	0.01	0.08	0.01	0.08
BSC GATE 5	0.01	0.01	0.01	0.08	0.01	0.08
GREEN 4	0.01	0.01	0.01	0.06	0.02	0.08
GTHQ	0.01	0.08	0.01	0.72	0.13	0.94

**Table 11:** Cancer risk of samples (adult).

Samples	Cd	Cr	Pb	As
ABH 3	3.1 × 10^−4^	1.10 × 10^−5^	5.06 × 10^−6^	1.7 × 10^−3^
DH 2	3.1 × 10^−4^	1.10 × 10^−5^	4.50 × 10^−6^	4.0 × 10^−4^
TGG 2	3.1 × 10^−4^	1.10 × 10^−5^	1.50 × 10^−6^	1.5 × 10^−4^
ETH 1	3.1 × 10^−4^	1.10 × 10^−5^	1.69 × 10^−6^	1.4 × 10^−3^
KKC 6	3.1 × 10^−4^	0.01 × 10^−5^	1.31 × 10^−6^	1.5 × 10^−3^
KKC 7	3.1 × 10^−4^	1.10 × 10^−5^	1.50 × 10^−6^	1.3 × 10^−3^
EH	3.1 × 10^−4^	1.10 × 10^−5^	4.31 × 10^−6^	1.8 × 10^−3^
AJ 3	3.1 × 10^−4^	1.10 × 10^−5^	3.19 × 10^−6^	1.4 × 10^−3^
BSC GATE 5	3.1 × 10^−4^	1.10 × 10^−5^	1.88 × 10^−6^	1.5 × 10^−3^
GREEN 4	3.1 × 10^−4^	1.10 × 10^−5^	2.06 × 10^−6^	1.2 × 10^−3^

**Table 12:** Cancer risk of samples (children).

	Cd	Cr	Pb	As
ABH 3	9.93 × 10^−4^	3.31 × 10^−5^	5.95 × 10^−4^	1.17 × 10^−3^
DH 2	9.93 × 10^−4^	3.31 × 10^−5^	5.30 × 10^−4^	2.43 × 10^−4^
TGG 2	9.93 × 10^−4^	3.31 × 10^−5^	1.76 × 10^−4^	9.71 × 10^−4^
ETH 1	9.93 × 10^−4^	3.31 × 10^−5^	1.98 × 10^−4^	9.05 × 10^−4^
KKC 6	9.93 × 10^−4^	9.93 × 10^−5^	1.55 × 10^−4^	1.06 × 10^−3^
KKC 7	9.93 × 10^−4^	3.31 × 10^−5^	1.76 × 10^−4^	8.83 × 10^−4^
EH	9.93 × 10^−4^	3.31 × 10^−5^	5.08 × 10^4^	1.24 × 10^−3^
AJ 3	9.93 × 10^−4^	3.31 × 10^−5^	3.75 × 10^−4^	9.48 × 10^−4^
BSC GATE 5	9.93 × 10^−4^	3.31 × 10^−5^	2.21 × 10^−4^	9.71 × 10^−4^
GREEN 4	9.93 × 10^−4^	3.31 × 10^−5^	2.43 × 10^−4^	7.95 × 10^−4^

## Data Availability

The data used to support the findings of this study are available from the corresponding author upon request.
